# Efficacy and Impact of Digital HIV Care Navigation in Young People Living With HIV in San Francisco, California: Prospective Study

**DOI:** 10.2196/18597

**Published:** 2020-05-08

**Authors:** Sean Arayasirikul, Caitlin Turner, Dillon Trujillo, Victory Le, Erin C Wilson

**Affiliations:** 1 San Francisco Department of Public Health Center for Public Health Research San Francisco, CA United States; 2 Department of Pediatrics University of California San Francisco San Francisco, CA United States; 3 Department of Psychiatry University of California San Francisco San Francisco, CA United States

**Keywords:** HIV/AIDS, digital HIV care navigation, young people living with HIV, mHealth

## Abstract

**Background:**

Young people are disproportionately impacted by HIV infection and exhibit poor HIV care continuum outcomes. Mobile health (mHealth) interventions are promising approaches to meet the unique needs of young people living with HIV. Youth-focused interventions are needed to improve HIV care continuum outcomes.

**Objective:**

This study assessed the preliminary efficacy and impact of a digital HIV care navigation intervention among young people living with HIV in San Francisco. Health electronic navigation (eNavigation or eNav) is a 6-month, text message–based, digital HIV care navigation intervention, in which young people living with HIV are connected to their own HIV care navigator through text messaging to improve engagement in HIV primary care.

**Methods:**

This study had a single-arm, prospective, pre-post design. The analysis included 120 young men who have sex with men or transwomen living with HIV aged between 18 and 34 years. We analyzed self-reported sociobehavioral information pre- and postintervention at baseline and 6 months, which was collected using computer-assisted self-interviewing surveys. We characterized the sample and built generalized estimating equation (GEE) models to assess differences in HIV care continuum outcomes at baseline and 6 months.

**Results:**

The characteristics according to the intervention completion status were not different from those of the overall sample. The mean age of the participants was 27.75 years (SD 4.07). Most participants (103/120, 85.8%) identified as men, and the sample was racially/ethnically diverse. At baseline, majority (99/120, 82.5%) of the participants had recently received primary HIV care, yet this was more likely in those who completed the intervention than in those who did not (54/60, 90% vs 45/60, 75%; χ^2^_1_=4.68, *P*=.03). More than half of the sample reported taking antiretroviral therapy (92/120, 76.7%) and having an undetectable viral load (65/120, 54.2%). The 6-month follow-up surveys were completed by 73.3% (88/120) of participants, and these participants were not characteristically different from the overall sample at baseline. GEE models indicated that participants had increased odds of viral suppression at 6 months as compared with baseline. No relevant additive or multiplicative interactions were noted on comparing outcome effects over time according to intervention completion.

**Conclusions:**

Digital HIV care navigation fills a critical gap in public health and HIV care systems, making these systems more responsive and accountable to the needs of the most vulnerable individuals. Our intervention bridges the time between primary care visits with interactive, tailored, personalized, and peer-delivered social support; information; and motivational interviewing to scaffold behavioral change. This study is part of the next wave of system-informed mHealth intervention research that will offer potentially disruptive solutions to traditional in-person delivered interventions and improve the health of the most vulnerable individuals.

**International Registered Report Identifier (IRRID):**

RR2-10.2196/16406

## Introduction

### Background

HIV remains a pressing public health issue, with over a million people in the United States living with HIV [1]. Although effective antiretroviral therapy (ART) is available to prevent HIV transmission from people living with HIV, disparities persist, and young people are affected. Even though the number of new infections among people aged 13 to 24 years decreased between 2010 and 2016, HIV transmission among young adults aged 25 to 34 years has increased [2]. Youth and young adults are not only disproportionately at risk for HIV but also experience relevant gaps in the HIV care continuum. Data from the Ryan White HIV/AIDS Program show that the viral suppression rate is less than 70% and is highest among those aged 25 to 30 years (68%), followed by those aged 13 to 18 years (66%) and aged 19 to 24 years (59%) [3]. Youth-focused interventions are needed to improve HIV care continuum outcomes.

Mobile health (mHealth) interventions have greatly increased over the last decade. A systematic review of systematic reviews identified 23 systematic reviews representing more than 10,000 scientific articles and involving close to 80,000 participants in 371 mHealth studies [4]. Yet, evidence for the efficacy and impact of mHealth interventions is unclear. This study assessed the preliminary efficacy and impact of a digital HIV care navigation intervention among young people living with HIV in San Francisco.

### Overview of Health Electronic Navigation

Health electronic navigation (eNavigation or eNav) is a 6-month, text message–based, digital HIV care navigation intervention, in which young people living with HIV are connected to their own HIV care navigator through text messaging to improve engagement in HIV primary care. The intervention includes delivery of the following: (1) HIV care navigation, (2) health promotion and education, (3) motivational interviewing, and (4) social support. HIV care navigation guides participants in knowing where, when, and how to access all health and related services, and increases access to appropriate resources (eg, primary medical care, mental health care, housing, insurance, and benefits) [5]. Health promotion and education ensure optimal health literacy for all participants by providing information on HIV biology, disease management, communication with providers, risk reduction, healthy behavior, and ART adherence. Motivational interviewing is a technique and a style of counseling that can help resolve the ambivalence that prevents patients from realizing their personal goals [6,7]. Social support is provided through the establishment of an open nonjudgmental care relationship between participants and their HIV care navigator to address life events and topics most important to young people living with HIV that may not be solely focused on their HIV care.

## Methods

### Ethics Approval

All procedures were in accordance with the ethical standards of the institutional and/or national research committee and with the 1964 Helsinki Declaration and its later amendments or comparable ethical standards. The study protocol was approved by the Institutional Review Board of the University of California, San Francisco (IRB #16-19675).

### Study Design and Recruitment

Data for the present analysis were collected from the Health eNav study at San Francisco Department of Public Health (2017-2018). Health eNav is a digital care navigation intervention designed to improve HIV care continuum outcomes among young men who have sex with men and transwomen living with HIV. A digital care navigator delivered the intervention via two-way SMS text messaging. This study had a single-arm, prospective, pre-post design. Study procedures are described in-depth in a prior manuscript [8].

Eligibility criteria for the study were as follows: identifying as a man who has sex with men or a transwoman; being between the ages of 18 and 34 years; and being newly diagnosed with HIV, not being engaged/retained in HIV care, or having a detectable viral load. Participants were recruited via convenience sampling from five clinics and community-based organizations in San Francisco serving young people living with HIV. If eligible, participants visited research staff at study offices within the San Francisco Department of Public Health, where informed consent was obtained. Of 170 individuals screened, 140 were eligible. However, 20 were subsequently lost to follow-up and were not enrolled. The final sample had 120 young men who have sex with men or transwomen living with HIV.

### Data Collection and Measures

Data for this analysis were collected using computer-assisted self-interviewing (CASI) surveys. Instruments collected self-reported sociobehavioral information pre- and postintervention at baseline and 6 months.

#### Sociodemographic Information

We assessed age at interview (in years), gender identity (transwoman vs man), race/ethnicity (non-Hispanic/Latinx, American Indian/Alaska Native, Asian, black/African American, multiracial, white, or Hispanic/Latinx), and education level (high school/General Educational Development [GED] or at least college education). We also examined the current living situation, defined as stable (owning or renting a house or apartment) or unstable (living with someone who owns or rents a home, living in temporary or transitional housing, or experiencing homelessness). Income was classified as earning US $0 to 250, US $251 to 600, US $601 to 1300, or US $1301 or more in the last month. Finally, we assessed history of incarceration.

#### Intervention Completion Status

We defined intervention completion as retention in the 6-month intervention. Among the 120 participants enrolled in the intervention, 60 were lost to follow-up and did not complete the 6-month intervention. The most common reason for loss to follow-up during the intervention was unknown (40/60, 67%), followed by participant phone loss (6/60, 10%), moving out of jurisdiction (9/60, 15%), incarceration (2/60, 3%), withdrawal (2/60, 3%), and death from causes unrelated to intervention participation (1/60, 2%). For participants who lost their phones, a maximum of one phone replacement was provided during the intervention. Participants who again lost their phones were unable to complete the intervention. The intervention completion status was dichotomously coded as “yes (1)” if participants completed the 6-month intervention and “no (0)” if they did not complete the intervention.

#### HIV Care Continuum Outcomes

Self-reported HIV care outcome data were collected using CASI surveys. In accordance with HIV care goals designated in 90-90-90 objectives [9], we dichotomized outcomes regarding whether participants received primary HIV care within the last 6 months (yes/no, 1/0), whether participants were taking ART (yes/no, 1/0), and whether participants were virally suppressed (eg, had a viral load of 200 copies/mL or less) (yes/no, 1/0).

### Statistical Analysis

Initially, we characterized the sample by describing baseline sociodemographics and self-reported HIV care continuum outcomes using baseline CASI data. Differences in baseline sociodemographics and outcomes on comparing participants who completed the intervention and those who did not were analyzed with bivariate statistical tests (chi-squared test or *t* test). As every participant received the digital care navigation intervention, we analyzed HIV care continuum outcome effects for all participants over the 6-month follow-up period. We produced three models using generalized estimating equations (GEEs) to account for within-subject correlations and to calculate the odds of HIV care continuum outcomes for the 6-month follow-up compared with baseline.

Finally, we assessed whether intervention completion modified HIV care continuum outcomes over time. To accomplish this, we tested for multiplicative and additive interactions between intervention completion and time (baseline or 6-month interview) and computed stratum-specific results. Initially, outcome odds or prevalences from the GEE models with interaction terms were converted to probabilities. We then calculated probability ratios for multiplicative interactions (ie, probability of having an undetectable viral load over the 6-month period for those who completed the intervention compared with those who did not complete the intervention), probability differences for additive interactions (ie, excess probability of having an undetectable viral load over the 6-month time period for those who completed the intervention compared with those who did not complete the intervention), and stratum-specific differences (ie, excess probability of having an undetectable viral load over the 6-month time period only among those who completed the intervention or only among those who did not complete the intervention). We reported statistically significant stratum-specific results and statistically significant interactions between intervention completion and time. All statistical analyses were performed using Stata 14 (StataCorp LLC College Station, Texas, USA) [10]; comparisons were considered statistically significant if the associated *P* value was less than .05.

## Results

Table 1 presents baseline sociodemographics and HIV care continuum outcomes for the Health eNav sample overall (n=120) and according to the intervention completion status. Except for incarceration and recent receipt of primary HIV care, characteristics according to the intervention completion status were not significantly different from those of the overall sample. The mean age of the participants was 27.75 years (SD 4.07). Most participants (103/120, 85.8%) identified as men. The sample was racially/ethnically diverse, with most participants identifying as Hispanic/Latinx, followed by white, multiple races, and black/African American, and few identifying as Asian or American Indian/Alaska Native. About half (68/120, 56.7%) of all participants completed some college education, yet most lived in unstable housing and had a monthly income of US $1300 or less. Recent incarceration was less likely in participants who completed the intervention than those who did not complete the intervention (11.67% vs. 26.67%, χ^2^_1_=4.36, *P*=.04).

In terms of baseline HIV care continuum outcomes, majority (99/120, 82.5%) of the participants had recently received primary HIV care, yet this was more likely in those who completed the intervention than in those who did not complete the intervention (54/60, 90% vs 45/60, 75%; χ^2^_1_=4.68, *P*=.03). More than half of the sample reported taking ART (92/120, 76.7%) and having an undetectable viral load (65/120, 54.2%) (Table 1).

The 6-month follow-up surveys were completed by 73.3% (88/120) of participants (Table 2), and these participants were not characteristically different from the overall sample at baseline. Table 2 presents the longitudinal results from the GEE models. After analyzing HIV care continuum outcomes over the 6-month study period, we observed that participants had increased odds of viral suppression at 6 months compared with baseline. We observed no statistically significant additive or multiplicative interactions on comparing outcome effects over time according to intervention completion. However, on testing for stratum-specific effects, we found that viral suppression increased over time among those who completed the intervention (83.89% probability of viral suppression at 6 months vs 69.60% probability of viral suppression at baseline; probability difference 14.29%, 95% CI 2.66%-26.41%). No corresponding stratum-specific difference in viral suppression was observed among those who did not complete the intervention.

**Table 1:** Differences in baseline sociodemographics and HIV care continuum outcomes among young men who have sex with men and transwomen overall and according to intervention completion (Health eNav, 2017-2019).

Characteristic			Overall, n (%) or mean (SD)	Did not complete the intervention, n (%) or mean (SD)	Completed the intervention, n (%) or mean (SD)	Group comparison	
						*t* test or chi-square test (df=1) statistic	*P* value
Total			120 (100.0)	60 (50.0)	60 (50.0)	—^a^	—
**Sociodemographics**							
	Age (years)		27.75 (4.07)	27.57 (4.09)	27.93 (4.07)	*t*=0.49	.62
	**Gender identity**					**χ^2^=0.07**	**.79**
		Transwoman	17 (14.2)	9 (15.0)	8 (13.3)		
		Man	103 (85.8)	51 (85.0)	52 (86.7)		
	**Race/ethnicity**					**χ^2^=4.42**	**.22**
		Black, non-Hispanic/Latinx	22 (18.3)	11 (18.3)	11 (18.3)		
		Hispanic/Latinx	38 (31.7)	24 (40.0)	14 (23.3)		
		Multiple races, non-Hispanic/Latinx	28 (23.3)	11 (18.3)	17 (28.3)		
		White, non-Hispanic/Latinx	32 (26.7)	14 (23.3)	18 (30.0)		
	**Education level**					**χ^2^=3.39**	**.07**
		High school/GED^b^ or less	52 (43.3)	31 (51.7)	21 (35.0)		
		Some college or more	68 (56.7)	29 (48.3)	39 (65.0)		
	**Current living situation**					**χ^2^=3.08**	**.08**
		Unstable	81 (67.5)	45 (75.0)	36 (60.0)		
		Stable	39 (32.5)	15 (25.0)	24 (40.0)		
	**Income in the last month (US $)**					**χ^2^=0.16**	**.98**
		601-1300	30 (25.0)	15 (25.0)	15 (25.0)		
		251-600	30 (25.0)	14 (23.3)	16 (26.7)		
		0-250	30 (25.0)	15 (25.0)	15 (25.0)		
		1301 or more	29 (24.2)	15 (25.0)	14 (23.3)		
	**Incarceration**					**χ^2^=4.36**	**.04**
		Yes	23 (19.2)	16 (26.7)	7 (11.7)		
		No	97 (80.8)	44 (73.3)	53 (88.3)		
**HIV care continuum outcomes**							
	**Received primary HIV care, last 6 months**					**χ^2^=4.68**	**.03**
		Yes	99 (82.5)	45 (75.0)	54 (90.0)		
		No	21 (17.5)	15 (25.0)	6 (10.0)		
	**Currently taking ART^c^**					**χ^2^=3.69**	**.06**
		Yes	92 (76.7)	42 (70.0)	50 (83.3)		
		No	27 (22.5)	18 (30.0)	9 (15.0)		
	**Undetectable viral load**					**χ^2^=0.13**	**.72**
		Yes	65 (54.2)	28 (46.7)	37 (61.7)		
		No	32 (26.7)	15 (25.0)	17 (28.3)		

^a^Not applicable.

^b^GED: General Educational Development.

^c^ART: antiretroviral therapy.

**Table 2:** Differences in HIV care continuum outcomes at baseline and 6 months among men who have sex with men and transwomen living with HIV (Health eNav, 2017-2019).

Characteristic			Baseline, n (%)	6 months, n (%)	Percentage mean change	Generalized estimating equation^a^ outcome effects over time	
						OR (95% CI)	*P* value
Total			120 (100.0)	88 (73.3)	—^b^	—	—
**HIV care continuum outcomes**							
	**Received primary HIV care, last 6 months**						
		No	21 (17.5)	9 (10.2)	—	Reference	—
		Yes	99 (82.5)	79 (89.8)	7.3	1.79 (0.82-3.93)	.14
	**Currently taking ART^c^**						
		No	27 (22.5)	16 (18.2)	—	Reference	—
		Yes	92 (76.7)	72 (81.8)	5.2	1.13 (0.70-1.83)	.61
	**Undetectable viral load**						
		No	32 (26.7)	13 (14.8)	—	Reference	—
		Yes	65 (54.2)	67 (76.1)	22.0	2.16 (1.30-3.57)	.003

^a^Three generalized estimating equation models were used for the analyses: (1) odds of receiving primary HIV care at 6 months compared with baseline, (2) odds of taking ART at 6 months compared with baseline, and (3) odds of being virally suppressed at 6 months compared with baseline.

^b^Not applicable.

^c^ART: antiretroviral therapy.

## Discussion

### Principal Findings

Our findings suggest that digital HIV care navigation is effective at promoting viral suppression at post-test compared with pretest. While many mHealth interventions developed for youth and young adults living with HIV are increasingly utilizing methods that leverage automated functionalities (eg, reminders, calendaring, gamification, and diary studies), Health eNav demonstrates the importance of real-time bidirectional interaction with an interventionist using text messaging. In other research, we found that digital HIV care navigation is feasible and acceptable and, in particular, responsive to the diverse needs of young people living with HIV in a metropolitan city with complex structural barriers [11]. Despite the lack of precedent, we argue that mHealth interventions must be scalable beyond the individual level in order to strengthen health systems and public health action [12]. While local health departments may be weary of integrating mHealth interventions into their system-wide approaches, this study provides evidence for the positive impact that such work can have on individuals in the health system [13].

### Limitations and Future Research

The findings should be interpreted with some limitations in mind. This study had a single-arm, prospective, pre-post design. Future studies using more robust study designs with randomization of participants to multiple study arms are needed to develop a cogent understanding of the short- and long-term efficacies of digital HIV care navigation. Additionally, future analyses that examine dose-response or employ just-in-time stepped wedge designs may offer more dynamic and responsive flexibility to the study population of young men who have sex with men and transwomen living with HIV. These individuals not only are the most vulnerable to HIV acquisition and the most likely to have poor HIV care continuum outcomes, but also face multiple structural barriers and complex stigma. While adolescence and young adulthood are already dynamic critical periods, for sexual and gender minorities living with HIV, the situation is exacerbated by intersectional stigma. More research on the understanding of how the critical axes of race, sexuality, gender identity, and HIV interact is needed. mHealth interventions addressing intersectional stigma must be developed.

### Conclusion

Despite the study limitations, digital HIV care navigation fills a critical gap in public health and HIV care systems, making these systems more responsive and accountable to the needs of the most vulnerable individuals. Our intervention bridges the time between primary care visits with interactive, tailored, personalized, peer-delivered social support; information; and motivational interviewing to scaffold behavioral changes, such as ART adherence. This study is part of the next wave of system-informed mHealth intervention research that will offer potentially disruptive solutions to traditional in-person delivered interventions and improve the health of the most vulnerable individuals. For ending the HIV epidemic in San Francisco, the United States, and globally, novel applications of low-tech hi-touch mHealth technologies, such as digital HIV care navigation, should be considered.

## Abbreviations

ART: antiretroviral therapy
CASI: computer-assisted self-interviewing
GEE: generalized estimating equation
mHealth: mobile health
